# What is *Tetramorium semilaeve* André, 1883? (Hymenoptera, Formicidae)

**DOI:** 10.3897/zookeys.512.10006

**Published:** 2015-07-06

**Authors:** Lech Borowiec, Christophe Galkowski, Sebastian Salata

**Affiliations:** 1 Department of Biodiversity and Evolutionary Taxonomy, University of Wrocław, Przybyszewskiego, 63/77, 51-148 Wrocław, Poland; 2 Route de Mounic, 33160 Saint-Aubin-de-Médoc, France

**Keywords:** Mediterranean Subregion, Crematogastrini, taxonomy, *Tetramorium semilaeve*

## Abstract

*Tetramorium semilaeve* André, 1883 is redescribed based on the type series and new material from terra typica (Pyrénées-Orientales). Lectotype worker is designated. Detailed descriptions of gyne and male are given. A review of material from the Mediterranean area suggests that in the past the name *Tetramorium semilaeve* has been applied to more than one species and the true *Tetramorium semilaeve* is common only in the western part of the Mediterranean basin. The structure of the male genitalia is the most reliable set of characters allowing a proper distinction of species in *Tetramorium semilaeve* species group. All names attributed to the former name “*semilaeve*” are discussed.

## Introduction

The genus *Tetramorium* Mayr, 1855 with 560 valid species and 21 valid subspecies is one of the most speciose ant genera of the subfamily Myrmicinae (Bolton in AntCat 2015). It is a genus with worldwide distribution in which the highest number of species has been recorded in the Afrotropical Region. 55 species (including 5 tramp, subcosmopolitan species) have been recorded so far from Europe and the Mediterranean basin (Borowiec 2014). Several of the names are poorly known, have not been revised since the original description, and are considered to be incertae sedis. Only *Tetramorium chefketi* and *Tetramorium ferox* groups have been recently revised and six other species have been lately redescribed in detail (Csösz and Markó 2004, Güsten et al. 2006, Csösz et al. 2007, 2014, Csösz and Schulz 2010, Steiner et al. 2010, Borowiec and Salata 2014, Espadaler and Goméz 2014) and molecular data suggests that some widely distributed species represent groups of cryptic taxa (Schlick-Steiner et al. 2006).

*Tetramorium semilaeve* André, 1883 was originally decribed as a variety of *Tetramorium caespitum*. No types were designated in the original description and André (1883) only noted that “Cette variété qui parait méridionale est répandue dans toute la region méditerranéenne de l’Europe, de l’Afrique et d’Asie” [This apparently southern variety is distributed throughout the entire region of the Mediterranean Europe, Africa, and Asia]. Emery (1891) was the first who note that André described this species based on material from Banyuls in Pyrénées Orientales (ex coll. Saulcy). Dalla Torre (1893), in his catalogue of Formicidae, treated this taxon as a good species and noted its distribution in southern Europe and northern Africa. Subsequently Forel (1902), based on material from Algeria, again reduced *semilaeve* to a race of *Tetramorium caespitum*. However, it is uncertain whether the specimens he mentions in this work were conspecific with *Tetramorium semilaeve* sensu André. Emery (1909) also considered *semilaeve* as a variety of *Tetramorium caespitum* and noted that its range cover an area of the whole Mediterranean basin and Central Asia. Eventually, Bondroit (1918) restored *Tetramorium semilaeve* a species rank and on the basis of material from André collection confirmed that type material came from Pyrénées-Orientales. The majority of subsequent authors accepted Bondroit’s proposition and the species has begun to be seen as a one of the most common member of the genus *Tetramorium* in the Mediterranean basin. Most of the populations of *Tetramorium* characterized by pale colour and incomplete head sculpture were treated as a different variants of this species with the result that more than twenty names are attributed to the taxon “*semilaeve*” sensu lato (see discussion).

Sanetra et al. (1999) based on electrophoretic studies were the first to suggest that the western and eastern populations of “*semilaeve*” complex represent two distinct taxa. Our morphological studies, especially the examination of male genitalia in samples from throughout the Mediterranean region, show that more than two species with characters so far attributed to *Tetramorium semilaeve* occur in this area. The access to the type specimens from André collection and the results of our study on the fresh material enabled us to answer questions regarding the real distribution of this species and determine characters distinguishing it from other taxa. In this work we designate the lectotype for *Tetramorium semilaeve* and prepare detailed redescription of all castes based on types and recently collected material from the terra typica (Pyrénées-Orientales).

## Material and methods

Specimens were compared using standard methods of comparative morphology. Photos were taken using a Nikon SMZ 1500 stereomicroscope, Nikon D5200 photo camera and Helicon Focus software.

All given label data are in their original spelling; a vertical bar (|) separates data on different rows and double vertical bar (||) separates labels.

**Figures 1–2.**
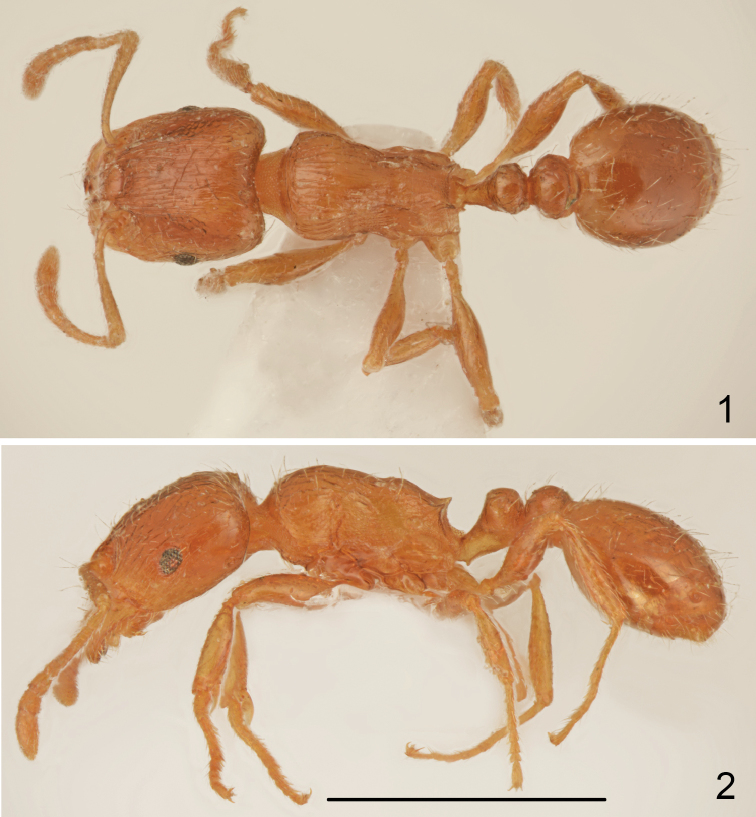
*Tetramorium semilaeve* André, lectotype **1** dorsal **2** lateral. Scale bar = 1 mm.

**Figures 3–4.**
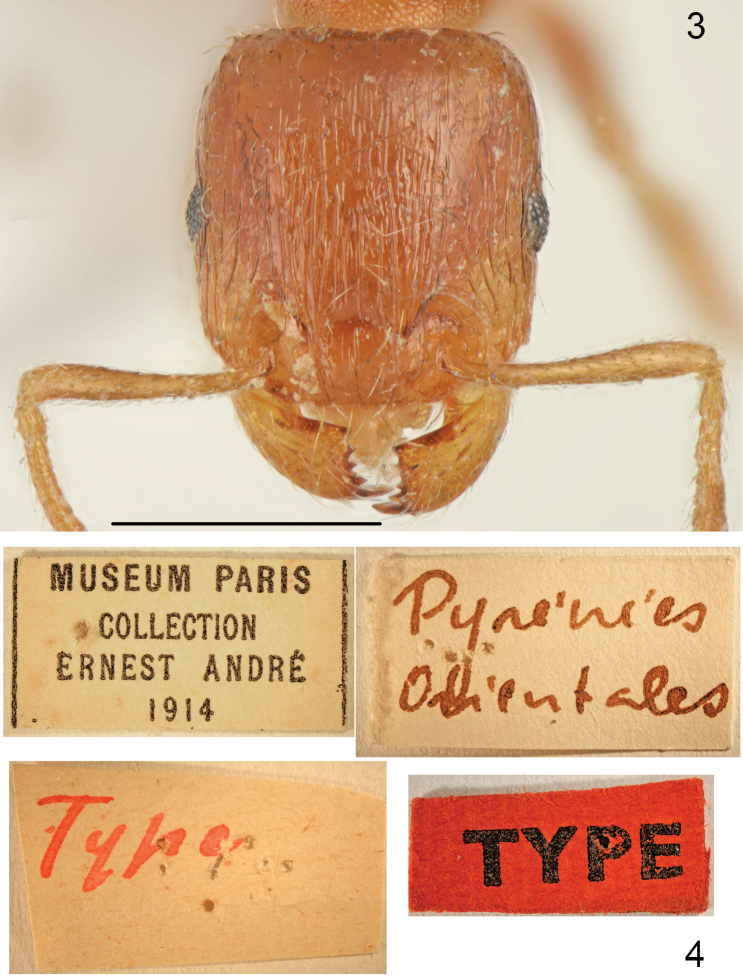
*Tetramorium semilaeve* André, lectotype **3** head **4** labels. Scale bar = 1 mm (**3**).

Our scheme of description corresponds with the revisions of *Tetramorium chefketi* and *Tetramorium ferox* groups (Csösz et al. 2007, Csösz and Schulz 2010: see Figs 1–4 on p. 4).

**Abbreviation of the depositories**:

CG coll. Christophe Galkowski, Saint-Aubin-de-Médoc, France;

DBET Department of Biodiversity and Evolutionary Taxonomy, University of Wrocław, Poland;

MNHN Muséum National d’Histoire Naturelle, Paris, France.

**Measurements and indices**:

CL length of head in full-face view, measured in a straight line from the anteriormost point of median clypeal margin to the mid-point of the posterior margin of the head. Concavity of posterior margin reduces CL;

CW maximum width of head in full-face view, including compound eyes;

CS cephalic size; calculated from the arithmetic mean of CL and CW. It is used as a less variable indicator of body size. For simplicity CS is used to describe body size;

EH the minimum diameter of the compound eye;

EL the maximum diameter of the compound eye;

EYE eye size index, calculated from the arithmetic mean of EL and EH, divided by CS;

OMD oculo-malar space. The minimal distance between anterior (lower) margin of the compound eye and the mandibular junction in profile;

FL the maximum distance between external borders of the frontal lobes;

FR the minimum width of the frons between the frontal carinae;

ML the diagonal length of mesosoma measured in lateral view from the anteriormost point of the pronotal slope to the posterior (or postero-ventral) margin of the propodeal lobes;

MW the maximum width of the pronotum from above;

NOH the maximum height of the petiolar node;

NOL the length of the petiolar node;

PEH the maximum height of the petiole in lateral view;

PEL the distance between the posteriormost point of the petiole and the petiolar spiracle;

PEW the maximum width of the petiole in dorsal view;

POC postocular distance. Measured from the reference line fitted on the posterior margin of compound eyes to median posterior margin of the head;

PPH the maximum height of the postpetiole in lateral view;

PPL the maximum length of the postpetiole in lateral view;

PPW the maximum width of the postpetiole in dorsal view;

SL the maximum length of the scape, measured from the proximal point of scape lobe to the distal end of scape;

SPL the minimum distance between the center of propodeal spiracle and the propodeal declivity;

SPSP the maximum length of propodeal teeth, measured in lateral view from the tip of spine to the propodeal spiracle;

WAIST (gyne only), waist index, calculated as (PEW+PPW)/CS.

## Descriptions

### Tetramorium semilaeve

Taxon classificationAnimaliaHymenopteraFormicidae

André, 1883

Tetramorium caespitum var. semilaeve André, 1883: 286 (terra typica: “toute la region méditerranéenne de l’Europe, de l’Afrique et d’Asie”).
Tetramorium semilaeve : Dalla Torre 1893: 134; Bondroit 1918: 109; Müller 1923: 104; Santschi 1927: 54.
Tetramorium caespitum r. semilaeve : Forel 1902: 148.
Tetramorium caespitum ssp. semilaeve : Emery 1909: 700, 703; Menozzi 1926: 182.

#### Type material examined.

Lectotype worker (here designated, no. ANTWEB1008880): Pyrénées | Orientales || Type || TYPE || MUSEUM PARIS | COLLECTION | ERNEST ANDRÉ | 1914 (MNHN); two paralectotype workers: Pyrénées-Orientales | (de Saulcy ||TYPE || Tetr. caespitum | race semilaeve || MUSEUM PARIS | COLLECTION | ERNEST ANDRÉ | 1914 (MNHN); three paralectotype workers: Pyr. | orient. | (d. Saulcy) || TYPE II T. | semilaeve || MUSEUM PARIS | COLLECTION | ERNEST ANDRÉ | 1914 (MNHN).

#### Other material examined.

*Pinned material*. 4 gynes, 6 workers: FRANCE, Pyrénées Orientales | Banyuls, Route du col, 196 m | 42.467 N / 3.141 E | 12 VI 2010, C. Galkowski || Collection L. Borowiec | Formicidae | LBC-FR00043 (DBET); 3 workers, 4 males: FRANCE, Pyrénées Orientales | Banyuls, Col de Séris, 290 m | 42.452 N / 3.141 E | 12 VI 2010, C. Galkowski || Collection L. Borowiec | Formicidae | LBC-FR00040 (DBET); 2 gynes, 3 workers, 3 males: FRANCE, Pyrénées Orientales | Banyuls, Col de Séris, 290 m | 42.452 N / 3.141 E | 12 VI 2010, C. Galkowski || Collection L. Borowiec | Formicidae | LBC-FR00041 (DBET); 4 gynes, 2 workers: FRANCE, Pyrénées Orientales | Banyuls, Col de Séris, 290 m | 42.452 N / 3.141 E | 12 VI 2010, C. Galkowski || Collection L. Borowiec | Formicidae | LBC-FR00042 (DBET); 1 worker: [FRANCE, Pyrénées Orientales ] Banyuls | Berland || MUSEUM PARIS | BANYULS-S-MER | PYRÉNÉES-ORIENTALES | L. BERLAND 1925 (MNHN); 3 gynes, 9 workers, 1 male: [FRANCE, Provence-Alpes-Côte d’Azur] Plan de Tour | 25.6.83 || F. BERNARD || MUSEUM PARIS (MNHN); 1 gyne: [FRANCE] CORSE – près Corte | Vallee moyenne du | Tavignano 11.VI.1976 I Rec. CASEVITZ-WEULERSSE II pris s/pierre *I* ecles au labo. | 17.VI.1976 || Museum Paris (MNHN).

*Alcohol material*. 10 workers: FRANCE, Pyrénées Orientales, Banyuls, Col de Séris, 42.452 N / 3.141 E, 12 VI 2010, leg. C. Galkowski (CG); 8 gynes, 6 workers: FRANCE, Pyrénées Orientales, Banyuls, Col de Séris no. 2, 42.452 N / 3.141 E, 12 VI 2010, leg. C. Galkowski (CG); 18 workers: FRANCE, Pyrénées Orientales, Banyuls, Col de Séris no. 3, 42.452 N / 3.141 E, 12 VI 2010, leg. C. Galkowski (CG);19 workers: FRANCE, Pyrénées Orientales, Banyuls, Cap Béar, 42.515 N / 3.132 E, 12 VI 2010, leg. C. Galkowski (CG); 27 workers: FRANCE, Pyrénées Orientales, Banyuls, Route du col, 42.467 N / 3.142 E, 12 VI 2010, leg. C. Galkowski (CG); 20 workers: FRANCE, Pyrénées Orientales, Banyuls, Paulilles, 42.501 N / 3.126 E, 12 VI 2010, leg. C. Galkowski (CG); 17 workers: FRANCE, Pyrénées Orientales, Banyuls, Bartissol, 42.481 N / 3.124 E, 12 VI 2010, leg. C. Galkowski (CG); 7 workers: FRANCE, Corsica, Bastia, 42.655 N / 9.449 E, IV 2011 (CG); 7 workers: FRANCE, Corsica, Piana, 42.231 N / 8.552 E, 23 VII 2011 (CG); 2 workers: SPAIN, Baleares, Mallorca, Cala D’Or, 14 m, 39,36666 N / 3,21666 E, 7 V 2009, leg. L. Borowiec (DBET); 2 workers: SPAIN, Baleares, Mallorca, Cala Egos, 11 m, 39,35 N / 3,21666 E, 7 V 2009, leg. L. Borowiec (DBET); 21 workers: SPAIN, Baleares, Mallorca, Cap de ses Salines from Punta de Mila to Punta Galera, 5 m, 39,26666 N / 3,03333 E, 9 V 2009, leg. L. Borowiec (DBET); 5 workers: SPAIN, Baleares, Mallorca, Parc Natural Mondrago n. Cala Egos, 12 m, 39,35 N / 3,18333 E, 11 V 2009, leg. L. Borowiec (DBET); 1 gyne, 7 workers, 4 males: SPAIN, Baleares, Mallorca, Cap de ses Salines from Punta de Mila to Punta de sa Cresta, 8 m, 39,26666 N / 3,06666 E, 12 V 2009, leg. L. Borowiec (DBET); 6 workers: SPAIN, Baleares, Mallorca, Ermita de Betlem n. Arta, 378 m, 39,71666 N / 3,31666 E, 12 V 2009, leg. L. Borowiec (DBET); 4 workers: SPAIN, Baleares, Mallorca, Colonia Sant Jordi, 4 m, 39,31666 N / 2,98333 E, 15 V 2009, leg. L. Borowiec (DBET); 4 workers: Spain, Andalucia, Malaga Pr., road Ojén-Refugio de Juanar, 544 m, 36,59358 N / 4,85621 W, 6 V 2014, leg. L. Borowiec (DBET); 5 workers: SPAIN, Andalucia, Malaga Pr., Igualeja, 720 m, 36,63259 N / 5,1179 W, 7 V 2014, leg. L. Borowiec (DBET); 4 workers: SPAIN, Andalucia, Malaga Pr., road Marbella-Istán, 145 m, 36,53324 N / 4,94905 W, 11 V 2014, leg. L. Borowiec; 50 workers: SPAIN, Andalucia, Cádiz Pr., nr. Getares, 36,06698 N / 5,44166 W, 8 V 2014, 21 m, leg. L. Borowiec (DBET); 5 workers: SPAIN, Andalucia, Cádiz Pr., road Tarifa-El Bujeo, 262 m, 36,05206 N / 5,55 W, 9 V 2014, leg. L. Borowiec (DBET); 10 workers: SPAIN, Andalucia, Cádiz Pr., Venta de Ojén, 248 m, 36,15910 N / 5,58684 W, 9 V 2014, leg. L. Borowiec (DBET); 20 workers: SPAIN, Catalonia, Alt Empordà, Cap de Creus n. Cadaques, 203 m 42°18N/3°13W, 2 IX 2011, leg. L. Borowiec (DBET); SPAIN, Catalonia, Alt Empordà, n. Llançà, 79 m, 42°21N/3°06W, 2 IX 2011, leg. L. Borowiec (DBET); 10 workers: ITALY, N Calabria, Scalea city-castle hill, 49 m, 39,81859 N/15,78963 E, 25 VIII 2014, leg. L. Borowiec (DBET); 5 workers: ITALY, N Calabria, n. Grisolia loc. 2, 484 m, 39,71887 N/15,88376 E, 2 IX 2014, leg. L. Borowiec (DBET); 4 workers: ITALY, N Calabria, n. Papasidero loc. 1, 162 m, 39,87390 N/15,90534 E, 5 IX 2014, leg. L. Borowiec; (DBET) 5 workers: ITALY, N Calabria, n. Tortora, 388 m, 39,94668 N/15,80452 E, 6 IX 2014, leg. L. Borowiec (DBET).

#### Redescription.

**Figures 5–6.**
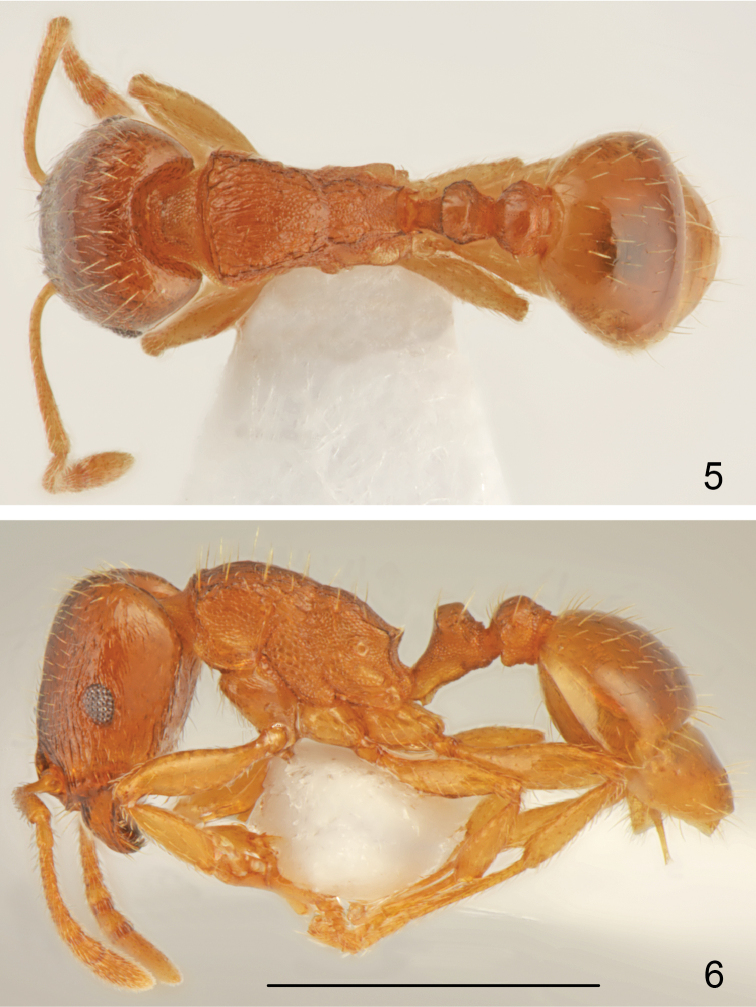
*Tetramorium semilaeve* André, worker **5** dorsal **6** lateral. Scale bar = 1 mm.

**Figures 7–9.**
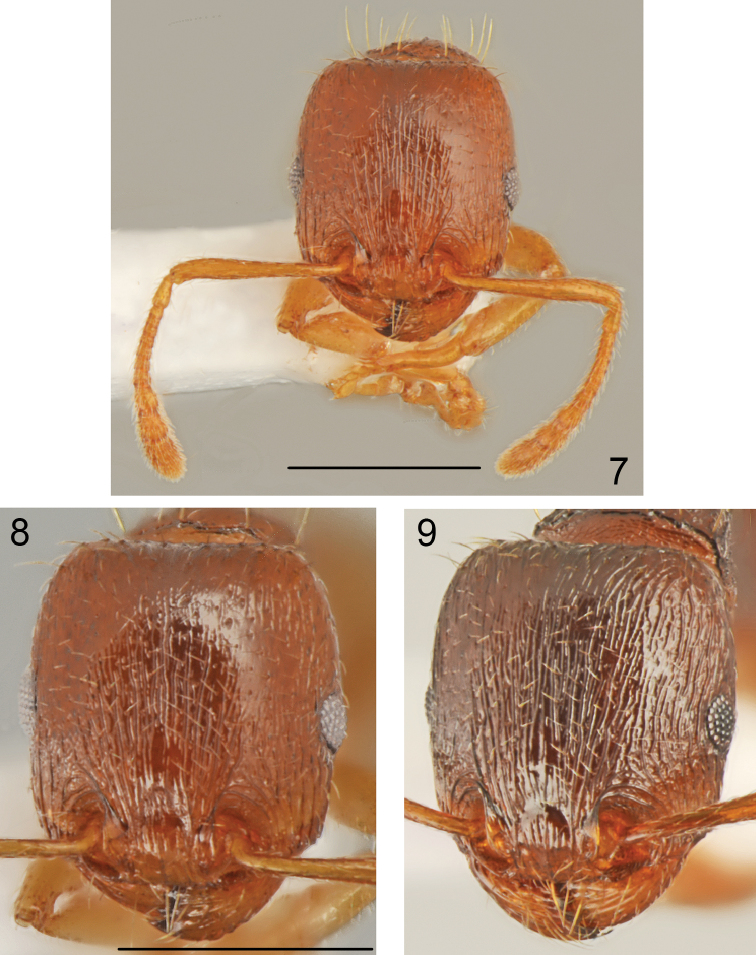
*Tetramorium semilaeve* André, worker **7** head and antennae **8** sculpture of head of the most common morphotype **9** sculpture of head of the most sculptured morphotype. Scale bar = 1 mm.

*Worker* (Figs 1–3, 5–9). Measurements and indices (n=15): CL: 0.723 ± 0.034 (0.637-0.771); POC: 0.293 ± 0.021 (0.246-.324); CW: 0.693 ± 0.037 (0.606-0.749); FR: 0.253 ± 0.015 (0.234-0.279); FL: 0.262 ± 0.017 (0.235-0.291); SL: 0.534 ± 0.03 (0.503-0.626); OMD: 0.18 ± 0.021 (0.145-0.223); EL: 0.13 ± 0.01 (0.106-0.145); EH: 0.091 ± 0.006 (0.078-0.101); ML: 0.781 ± 0.043 (0.737-0.894); SPSP: 0.133 ± 0.015 (0.112-0.179); SPL: 0.099 ± 0.007 (0.089-0.112); PEL: 0.184 ± 0.01 (0.167-0.201); NOL: 0.144 ± 0.013 (0.128-0.168); PPL: 0.176 ± 0.009 (0.156-0.19); PEH: 0.239 ± 0.018 (0.212-0.291); NOH: 0.158 ± 0.017 (0.14-0.218); PPH: 0.228 ± 0.02 (0.201-0.291); MW: 0.45 ± 0.027 (0.413-0.508); PEW: 0.22 ± 0.014 (0.201-0.246); PPW: 0.256 ± 0.018 (0.223-0.307); CS: 0.707 ± 0.036 (0.622-0.76); EYE: 0.155 ± 0.007 (0.143-0.166); CL/CW: 1.042 ± 0.013 (1.015-1.075); FR/CS: 0.358 ± 0.01 (0.345-0.378); FL/FR: 1.025 ± 0.02 (0.996-1.066); SL/CS: 0.756 ± 0.03 (0.732-0.841); MW/CS: 0.636 ± 0.018 (0.612-0.682); PEW/PPW: 0.861 ± 0.033 (0.801-0.918); NOH/NOL: 1.1 ± 0.113 (0.929-1.298); NOH/PEL: 0.86 ± 0.085 (0.819-1.085); NOL/PEL: 0.784 ± 0.045 (0.705-0.871); PEH/NOL: 1.66 ± 0.152 (1.399-1.922); PEW/PEH: 0.926 ± 0.049 (0.805-1.0); CS/PEW: 3.215 ± 0.089 (3.026-3.336); CS/PPW: 2.769 ± 0.154 (2.425-3.024); CW/MW: 1.541 ± 0.046 (1.435-1.602);

Small to medium size, CS: 0.707 [0.622-0.76]. In most specimens whole body pale yellowish-brown and appendages yellow, the palest specimens completely yellow, the darkest specimens yellowish-brown but never dark brown or black. Head nearly square CL/CW: 1.042 [1.015-1.075], with almost parallel sides, straight or slightly concave occipital margin and narrowly rounded occipital corners. Eyes small, EYE 0.155 [0.143-0.166]. Frons moderately wide, FR/CS 0.358 [0.345-0.378], frontal lobes as wide as frons, FL/FR: 1.025 [0.996-1.066]. Scape short, SL/CS 0.756 [0.732-0.841], without dorsal carina basally, surface smooth and shiny. Promesonotal dorsum slightly convex, metanotal groove shallow, but distinct. Propodeal teeth short, spiniform, apex of spine located approximately at 2/3 height of mesosoma. Dorsal surface of petiole flat to slightly convex, NOH/NOL 1.1 [0.929-1.298], petiole relatively high, PEH/NOL 1.66 [1.399-1.922], postpetiole distinctly transverse (Figs 1, 5). General appearance finely rugose, ground surface shiny. Head dorsum partly longitudinally rugose and shiny between rugae, rugae extend occipital margin of head, occiput mostly smooth and shiny, sides in anterior half longitudinally rugose and shiny between rugae. In most specimens between frontal rugose area and rugosities along ocular area on each side runs longitudinal band without rugosities (Fig. 8) but smooth area never exceeds 1/5 of the anterior surface of head; in extremely sculptured specimens almost whole frontal surface of head with long rugae with very small smooth patch between interrupted rugae and occiput with fine rugosities (Fig. 9), dark coloured specimens usually have more distinct sculpture than pale coloured specimens but strongly sculptured and completely yellow specimens were also observed. Alitrunk dorsum rugose and microreticulate but never reticulate, only occasionally rugae on pronotum partly interrupted with indistinct microreticulation but pronotum never with smooth and shiny areas (Figs 1, 5). Sides of pronotum and meso- and metapleuron usually coarsely microreticulate, sometimes reticulation tends to form transverse lines but surface never appears striate or rugose (Figs 2, 6). Dorsum of petiolar node smooth and shiny with sides carinate, lateral surface microreticulate. Dorsum of postpetiole smooth and shiny, sides microreticulate. First gastral tergite smooth and shiny. Whole dorsum, including head, covered with sparse setae, the longest on pronotum and the shortest on frons. Ventral surface of head with sparse short and 2-3 moderately long setae not forming a psammophore.

**Figures 10–11.**
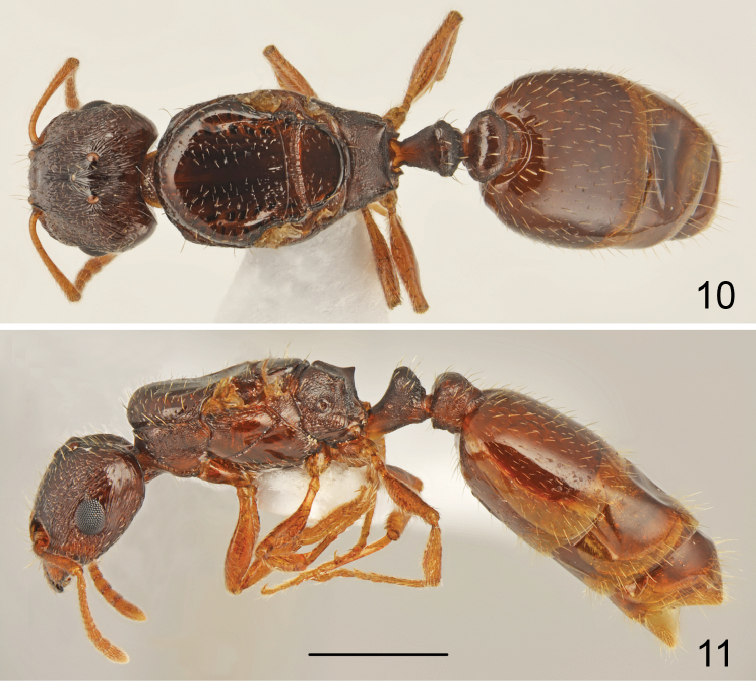
*Tetramorium semilaeve* André, gyne **10** dorsal **11** lateral. Scale bar = 1 mm.

**Figure 12–13.**
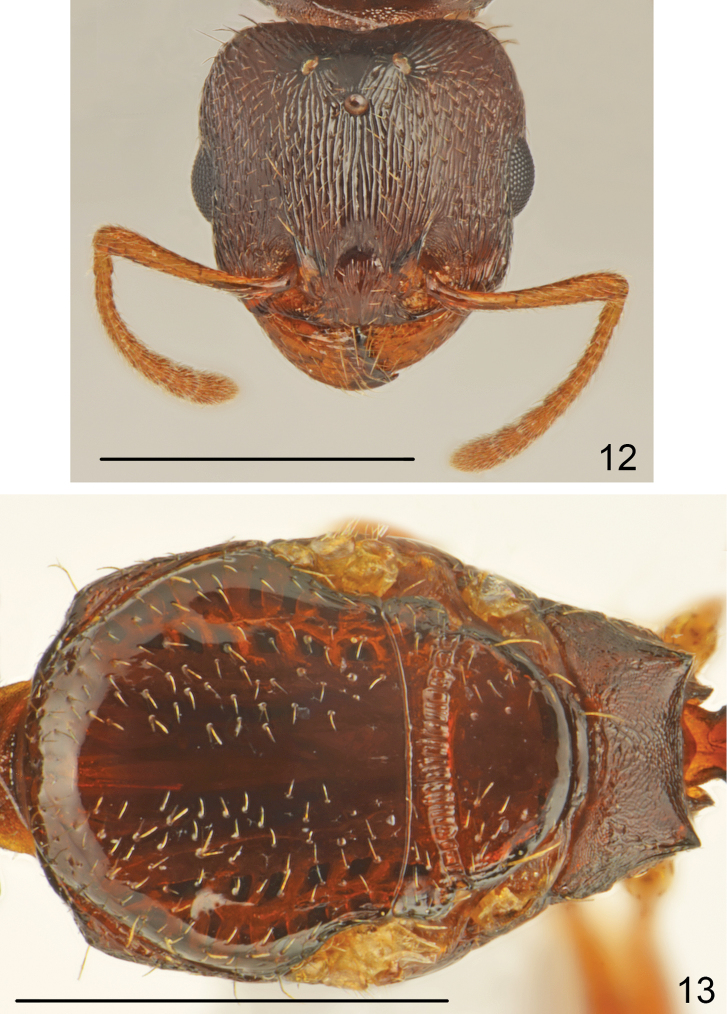
*Tetramorium semilaeve* André, gyne **12** head **13** mesosoma dorsal. Scale bar = 1 mm.

*Gyne* (Figs 10–13). Measurements and indices (n=11): CL: 0.999 ± 0.015 (0.983-1.027); POC: 0.378 ± 0.019 (0.34-.413); CW: 1.09 ± 0.05 (0.978-1.161); FR: 0.397 ± 0.009 (0.38-0.412); FL: 0.385 ± 0.018 (0.357-0.413); SL: 0.715 ± 0.015 (0.693-0.737); OMD: 0.223 ± 0.016 (0.212-0.257); EL: 0.274 ± 0.01 (0.257-0.291); EH: 0.218 ± 0.012 (0.193-0.235); ML: 1.699 ± 0.217 (1.053-1.813); SPSP: 0.267 ± 0.015 (0.24-0.291); SPL: 0.193 ± 0.01 (0.173-0.201); PEL: 0.298 ± 0.014 (0.279-0.324); NOL: 0.225 ± 0.022 (0.179-0.256); PPL: 0.301 ± 0.01 (0.285-0.313); PEH: 0.424 ± 0.011 (0.408-0.447); NOH: 0.283 ± 0.019 (0.257-0.313); PPH: 0.423 ± 0.017 (0.391-0.447); MW: 1.069 ± 0.04 (1.0-1.141); PEW: 0.393 ± 0.022 (0.348-0.419); PPW: 0.509 ± 0.019 (0.48-0.547); CS: 1.046 ± 0.032 (0.986-1.094); EYE: 0.235 ± 0.012 (0.219-0.251); CL/CW: 0.917 ± 0.038 (0.882-0.985); FR/CS: 0.377 ± 0.011 (0.36-0.393); FL/FR: 0.978 ± 0.035 (0.913-1.027); SL/CS: 0.686 ± 0.019 (0.654-0.717); MW/CS: 1.024 ± 0.043 (0.94-1.088); PEW/PPW: 0.773 ± 0.035 (0.692-0.821); NOH/NOL: 1.266 ± 0.136 (1.094-1.587); NOH/PEL: 0.949 ± 0.053 (0.883-1.036); NOL/PEL: 0.755 ± 0.059 (0.63-0.847); PEH/NOL: 1.9 ± 0.175 (1.746-2.374); PEW/PEH: 0.931 ± 0.048 (0.818-0.971); CS/PEW: 2.62 ± 0.123 (2.475-2.879); CS/PPW: 2.052 ± 0.085 (1.947-2.185); CW/MW: 1.02 ± 0.057 (0.911-1.129); WAIST: 0.871 ± 0.035 (0.817-0.918).

Moderate size, CS 1.046 [0.986-1.094]. Whole body brown, appendages yellowish. Head wider than long, CL/CW 0.917 [0.882-0.985], with straight subparallel sides, shallowly emarginate occipital margin and narrowly rounded occipital corners. Frons moderately wide, FR/CS 0.377 [0.36-0.393], frontal lobes as wide as frons, FL/FR 0.978 [0.913-1.027]. Scape short, SL/CS 0.686 [0.654-0.717], without dorsal carina basally, smooth and shiny. Head as wide as scutum, MW/CS 1.024 [0.94-1.088]. Propodeal teeth very short. Dorsal crest of petiolar node in frontal view straight. Petiolar node dorsum steeply rounded backward. Petiole and postpetiole relatively narrow, WAIST 0.871 [0.817-0.918]. General appearance smooth and shiny. Head dorsum, occiput and sides rugulose, ground surface shiny or indistinctly microreticulate. Frons longitudinally rugulose (Fig. 12). Mesosoma flat, pronotum visible from above. Scutum and scutellum punctate along sides, in most specimens smooth and shiny (Fig. 13), only occasionally scutellum in corners with very short rugae and scutum in basal part with thin longitudinal rugae never extending to half length of scutum. Sides of pronotum ruguloso-reticulate and feebly microreticulate, anepisternum mostly smooth and shiny only in dorsal part with fine longitudinal rugae, katepisternum smooth and shiny. Dorsum of petiolar node distinctly reticulate and smooth, posterior surface granulate and more or less transversely rugose. Postpetiole distinctly transverse, sides narrowly rounded to subangulate (Fig. 10), dorsum of postpetiole smooth, sides granulate. First gastral tergite smooth and shiny. Whole dorsum, including head, covered with short, sparse setae. Ventral surface of head with several short setae, as long as to 1.5 times longer than frontal setae, arising posteriorly to buccal cavity.

**Figure 14–15.**
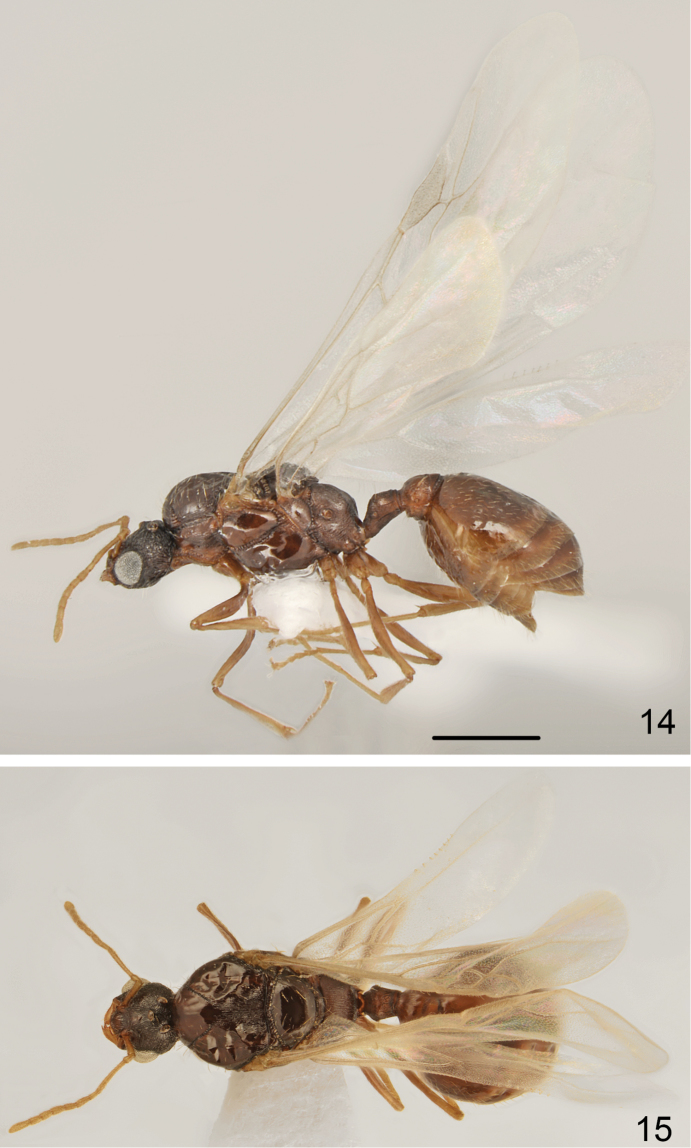
*Tetramorium semilaeve* André, male **14** lateral **15** dorsal. Scale bar = 1 mm.

**Figure 16–19.**
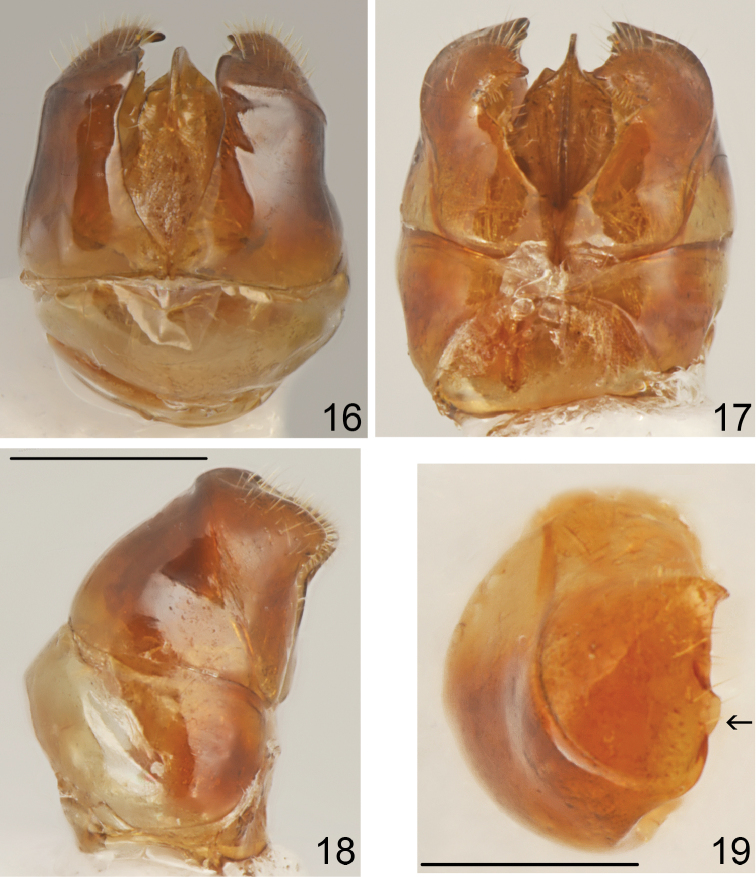
*Tetramorium semilaeve* André, male genitalia **16** dorsal **17** ventral **18** lateral **19** apex of paramere in dorsal view. Scale bar = 0.5 mm (**16–18**); 0.3 mm (**19**).

*Male* (Figs 14–19). Measurements and indices (n=10): CL: 0.667 ± 0.018 (0.637-0.693); POC: 0.279 ± 0.019 (0.263-.313); CW: 0.807 ± 0.024 (0.771-0.827); FR: 0.238 ± 0.012 (0.221-0.257); FL: 0.292 ± 0.013 (0.277-0.307); SL: 0.333 ± 0.016 (0.307-0.358); OMD: 0.068 ± 0.016 (0.056-0.089); EL: 0.312 ± 0.01 (0.302-0.324); EH: 0.251 ± 0.011 (0.235-0.263); ML: 1.716 ± 0.069 (1.626-1.785); SPSP: 0.226 ± 0.014 (0.212-0.246); SPL: 0.205 ± 0.013 (0.184-0.223); PEL: 0.275 ± 0.017 (0.257-0.302); NOL: 0.176 ± 0.018 (0.156-0.201); PPL: 0.287 ± 0.017 (0.263-0.302); PEH: 0.286 ± 0.022 (0.257-0.313); NOH: 0.163 ± 0.01 (0.156-0.179); PPH: 0.418 ± 0.015 (0.391-0.425); MW: 1.125 ± 0.056 (1.056-1.223); PEW: 0.337 ± 0.023 (0.307-0.368); PPW: 0.478 ± 0.022 (0.453-0.503); CS: 0.737 ± 0.019 (0.704-0.755); EYE: 0.382 ± 0.009 (0.37-0.395); CL/CW: 0.826 ± 0.023 (0.797-0.857); FR/CS: 0.323 ± 0.012 (0.314-0.345); FL/FR: 1.228 ± 0.06 (1.132-1.306); SL/CS: 0.452 ± 0.015 (0.436-0.478); MW/CS: 1.528 ± 0.093 (1.411-1.684); PEW/PPW: 0.705 ± 0.04 (0.668-0.767); NOH/NOL: 0.928 ± 0.076 (0.812-1.0); NOH/PEL: 0.595 ± 0.059 (0.517-0.668); NOL/PEL: 0.643 ± 0.069 (0.559-0.75); PEH/NOL: 1.625 ± 0.114 (1.502-1.788); PEW/PEH: 1.182 ± 0.061 (1.1-1.284); CS/PEW: 2.216 ± 0.165 (2.019-2.438); CS/PPW: 1.558 ± 0.085 (1.443-1.652); CW/MW: 0.719 ± 0.045 (0.639-0.783)

Whole body dark brown, appendages yellowish. Head with feebly convex sides, slightly rounded occipital margin and widely rounded occipital corners. Scutum distinctly wider than head. Propodeum rounded in profile or with only indistinct angulation in position of propodeal teeth. Dorsal crest of petiolar node with obtuse transversal edge, slightly emarginated in frontal view. Head distinctly granulate, dull, scutum in anterior part and laterally smooth and shiny, rest microreticulate. Scutellum ostly smooth and shiny, at corners microreticulate and in anterior part with thin transverse rugosities. Sides of alitrunk smooth and shiny. Dorsum of petiolar node microgranulate and microreticulate, dull, postpetiole mostly microreticulate only top partly smooth and shiny. First gastral tergite smooth and shiny. Male genitalia stout (Figs 16–18), in lateral view only slightly constricted before apex with obtuse inner angle, top shortly and sparsely pubescent, ventral and dorsal margins of parameres shallowly incised, top of inner margin of paramere before apical denticle straight with dentiform plate distinctly clearly extending beyond the sharp edge of paramere (Fig. 19, arrow indices this plate).

#### Diagnosis.

*Tetramorium semilaeve* belongs to the group of Mediterranean species with workers pale-coloured, from yellow to yellowish-brown but never dark brown or black, head sculpture partly reduced, at least with postocular area and sides of frons without distinct striation but with at least half of the surface of the anterior part of head striate, striae on top of head not diverging laterally, pronotum mostly striate, sides of mesosoma only microreticulate and microgranulate without carinae, short propodeal spines, petiole and postpetiole dorsally with polished area, and first abdominal tergite smooth and shiny, without microreticulation or striation; gyne with flat mesonotal plate, without a distinct striation or the striation is indistinct, short not extending behind half length of the plate, scutellum without striation or only on sides with short, indistinct striae, postpetiole not enlarged; male genitalia stout, in lateral view only slightly constricted before apex, ventral and dorsal margins of parameres shallowly incised, top of inner margin of paramere before apical denticle straight with dentiform plate distinctly clearly extending beyond the sharp edge of paramere (Fig. 19, arrow indices this plate). The presence of dentiform plate on top of the inner margin of paramere is the best character distinguishing *Tetramorium semilaeve* from all other species related to this taxon.

#### Biological data.

*Tetramorium semilaeve* prefers very warm and dry places. Most observed nests were located in the littoral zone on the flat, sandy areas covered with sparse vegetation or on stony pastures. The locality with the highest altitude in the examined material here is from Andalucia, Igualeja placed 720 m a.s.l. Sanetra et al. (1999) noted that in Italy most samples were collected below 1000 m a.s.l. and only occasionally above this altitude. All nests were located under stones, from small to very large size, and consisted of from several to several hundred workers. Because gynes were rarely collected, Sanetra et al. (1999) suggested that this species is monogynous but we observed more than one gyne in one nest in five cases. The following ant species were recorded in the same areas as *Tetramorium semilaeve*:

France, Banyuls, Col de Séris: *Camponotus aethiops* (Latreille), *Lasius myops* Forel, *Pheidole pallidula* (Nylander), *Plagiolepis pygmaea* (Latreille), *Temnothorax recedens* (Nylander), *Tetramorium* sp.;

France, Banyuls, Cap Béar: *Aphaenogaster senilis* Mayr, *Pheidole pallidula* (Nylander), *Plagiolepis pygmaea* (Latreille), *Tapinoma nigerrimum* (Nylander), *Temnothorax niger* (Forel), *Tetramorium* sp.;

France, Banyuls, Route du col: *Cataglyphis piliscapa* (Forel), *Lasius lasioides* (Emery), *Pheidole pallidula* (Nylander), *Plagiolepis pygmaea* (Latreille), *Solenopsis fugax* (Latreille), *Tetramorium* sp.;

France, Banyuls, Paulilles: *Aphaenogaster senilis* Mayr, *Iberoformica subrufa* (Roger), *Pheidole pallidula* (Nylander), *Plagiolepis pygmaea* (Latreille), *Tapinoma nigerrimum* (Nylander), *Temnothorax kutteri* (Cagniant ), *Temnothorax niger* (Forel), *Tetramorium* sp.;

France, Corsica, Bastia: *Aphaenogaster spinosa* Emery, *Crematogaster scutellaris* (Olivier), *Formica cunicularia* Latreille, *Pheidole pallidula* (Nylander), *Plagiolepis pygmaea* (Latreille), *Temnothorax exilis* (Emery), *Tetramorium* sp.;

France, Corsica, Piana: *Aphaenogaster spinosa* Emery, *Formica cunicularia* Latreille, *Messor minor* (André), *Messor wasmanni* Krausse, *Plagiolepis pygmaea* (Latreille), *Solenospis fugax* (Latreille), *Tapinoma nigerrimum* (Nylander), *Tetramorium* sp., *Tetramorium meridionale* Emery;

Mallorca, Cap de ses Salines from Punta de Mila to Punta de sa Cresta: *Crematogaster laestrygon* Emery, *Plagiolepis pygmaea* (Latreille), *Plagiolepis schmitzii* Forel, *Temnothorax* sp.;

Mallorca, Cap de ses Salines from Punta de Mila to Punta Galera: *Camponotus ruber* Emery, *Crematogaster auberti* Emery, *Lasius lasioides* (Emery), *Plagiolepis pygmaea* (Latreille), *Plagiolepis schmitzii* Forel;

Mallorca, Cala D’Or: Messor cf. structor;

Mallorca, Parc Natural Mondrago: *Camponotus ruber* Emery, *Crematogaster laestrygon* Emery, *Linepithema humile* (Mayr), *Messor bouvieri* Bondroit, Messor cf. structor, *Plagiolepis pygmaea* (Latreille);

Mallorca, Ermita de Betlem n. Arta: *Crematogaster auberti* Emery, *Crematogaster laestrygon* Emery, *Crematogaster scutellaris* (Olivier), *Lasius grandis* Forel, *Lasius lasioides* (Emery), *Messor bouvieri* Bondroit, *Pheidole pallidula* (Nylander), *Plagiolepis pygmaea* (Latreille), *Plagiolepis xene* Stärcke, *Tapinoma madeirense* Forel, *Temnothorax algiricus* Forel, *Temnothorax recedens* (Nylander);

Mallorca, Colonia Sant Jordi: *Linepithema humile* (Mayr), *Messor bouvieri* Bondroit, Messor cf. structor, *Monomorium salomonis* (Linnaeus), *Temnothorax* sp.;

Andalucia, road Ojén-Refugio de Juanar: *Aphaenogaster gibbosa* (Latreille), *Camponotus foreli* Emery, *Camponotus pilicornis* (Roger), *Iberoformica subrufa* (Roger), *Plagiolepis schmitzii* Forel, *Tapinoma nigerrimum* (Nylander);

Andalucia, road Marbella-Istán: *Camponotus foreli* Emery, *Crematogaster auberti* Emery, *Crematogaster sordidula* (Nylander), *Iberoformica subrufa* (Roger), *Monomorium salomonis* (Linnaeus), *Plagiolepis schmitzii* Forel;

Andalucia, nr. Getares: *Anochetus ghilianii* (Spinola), *Camponotus barbaricus* Emery, *Crematogaster auberti* Emery, *Goniomma hispanicum* (André), *Messor barbarus* (Linnaeus), *Tapinoma nigerrimum* (Nylander), Temnothorax cf. flavispinus;

Andalucia, road Tarifa-El Bujeo: *Anochetus ghilianii* (Spinola), *Aphaenogaster senilis* Mayr, *Camponotus barbaricus* Emery, *Camponotus gestroi*, *Messor barbarus* (Linnaeus), *Pheidole pallidula* (Nylander), *Tapinoma nigerrimum* (Nylander), Temnothorax cf. flavispinus;

Andalucia, Venta de Ojén: *Camponotus cruentatus* (Latreille), *Cataglyphis iberica* (Emery), Temnothorax cf. luteus;

Calabria, Scalea city-castle hill: *Aphaenogaster campana* Emery, *Camponotus piceus* (Leach), *Hypoponera eduardi* (Forel), *Lasius emarginatus* (Olivier), *Lepisiota frauenfeldi* (Mayr), *Linepithema humile* (Mayr), *Messor capitatus* (Latreille), *Messor wasmanni* Krausse, *Pheidole pallidula* (Nylander), *Plagiolepis taurica* Santschi, *Tapinoma nigerrimum* (Nylander);

Calabria, n. Grisolia loc. 2: *Aphaenogaster campana* Emery, *Bothriomyrmex communistus* Santschi, *Camponotus aethiops* (Latreille), *Camponotus lateralis* (Olivier), *Camponotus piceus* (Leach), *Crematogaster scutellaris* (Olivier), *Lasius emarginatus* (Olivier), *Lasius myops* Forel, *Messor capitatus* (Latreille), *Pheidole pallidula* (Nylander), *Plagiolepis pygmaea* (Latreille), *Plagiolepis xene* Stärcke, *Solenopsis fugax* Latreille, *Temnothorax exilis* (Emery), *Temnothorax flavicornis* (Emery), *Temnothorax leviceps* (Emery);

Calabria, n. Papasidero loc. 1: *Aphaenogaster campana* Emery, *Camponotus aethiops* (Latreille), *Camponotus dalmaticus* (Nylander), *Camponotus lateralis* (Olivier), *Camponotus nylanderi* Emery, *Crematogaster scutellaris* (Olivier), *Crematogaster sordidula* (Nylander), *Lasius emarginatus* (Olivier), *Messor capitatus* (Latreille), *Messor wasmanni* Krausse, *Pheidole pallidula* (Nylander), *Plagiolepis pygmaea* (Latreille), *Temnothorax flavicornis* (Emery), *Temnothorax leviceps* (Emery), *Temnothorax recedens* (Nylander);

Calabria, n. Tortora: *Aphaenogaster campana* Emery, *Camponotus lateralis* (Olivier), *Camponotus nylanderi* Emery, *Crematogaster scutellaris* (Olivier), *Lasius emarginatus* (Olivier), *Lasius myops* Forel, *Messor wasmanni* Krausse, *Pheidole pallidula* (Nylander), *Plagiolepis pygmaea* (Latreille), *Temnothorax leviceps* (Emery), *Temnothorax lichtensteini* (Bondroit), *Temnothorax recedens* (Nylander).

**Figure 20.**
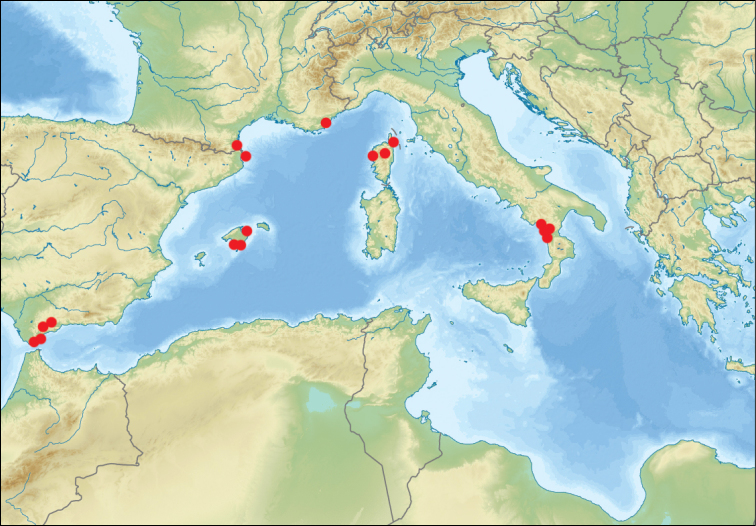
*Tetramorium semilaeve* André, revised localities.

## Discussion

Numerous names of various taxonomic rank have been attributed to the taxon “*semilaeve*”. Below we listed all these names with comments (tt = terra typica):

Tetramorium caespitum r. depressum Forel, 1892: 455; as a form of *Tetramorium semilaeve*: Santschi, 1936: 202 (tt: Canary Islands) – this taxon is common in the Canary Islands and was also recorded from north-western Africa usually as subspecies of *Tetramorium semilaeve* (Cagniant 1997). Espadaler (2007) raised this name to a species rank and noted that *Tetramorium semilaeve* and *Tetramorium depressum* differ in head sculpture and male genitalia. We have examined several workers from Tenerife and agree with that but we are not sure whether the reports from north-western Africa relate to the same species. Our material from the Mediterranean suggests that more than one species with reduced head sculpture occurs in this area;

Tetramorium caespitum subsp. judas Wheeler & Mann, 1916: 172; as subsp. of *Tetramorium semilaeve*: Menozzi, 1933: 12 (tt: Palestine) – our materials showed than none of the populations from the Near East are conspecific with the true *Tetramorium semilaeve* but we are not sure how many species occur in this region due to absence of a sufficient number of samples with males and gynes;

*Tetramorium hippocratis* Agosti & Collingwood, 1987: 56 (= Tetramorium caespitum semilaeve var. hippocratis Emery, 1921: 217) (tt: Turkey, unavailable name) – we examined syntype images available on AntWeb (Available from: https://www.antweb.org/specimen/CASENT0904822) of this taxon and in our opinion it represents a distinct species more similar to *Tetramorium sahlbergi* Forel than to *Tetramorium semilaeve* André;

Tetramorium caespitum st. semilaeve var. ernesti Santschi, 1921a: 431 (tt: France and Algeria) – unavailable name;

Tetramorium caespitum st. semilaeve var. romana Santschi, 1921a: 431 (tt: ?) – unavailable name;

Tetramorium caespitum subsp. semilaeve var. fortunatarum Emery, 1925: 190 (tt: Canary Islands) – although Hohmann et al. (1993) treated this name as a subspecies of *Tetramorium semilaeve*, in accordance with the Code of Zoological Nomenclature it remains an unavailable name, likely conspecific with *Tetramorium depressum*;

Tetramorium caespitum subsp. semilaeve var. palmense Wheeler, 1927: 113 (tt: Canary Islands) – unavailable name, likely conspecific with *Tetramorium depressum*;

Tetramorium semilaeve var. lipareum Santschi, 1927: 55 (tt: Lipari and Sicily Islands) – Sanetra et al. (1999) studied syntypes of this name and synonymized it with *Tetramorium punctatum* (see comments below);

Tetramorium semilaeve var. punctatum Santschi, 1927: 55 (tt: Sicily) – Sanetra et al. (1999) studied syntypes of this name, designated lectotype and raised this taxon to species rank based on the morphology of workers, gynes and electyrophoretic study. We agree with them, both taxa differ also in structure of male genitalia;

Tetramorium semilaeve var. siciliense Santschi, 1927: 56 (tt: Sicily) – Sanetra et al. (1999) based on syntypes synonymized this name with *Tetramorium semilaeve*. We agree with them, in Sicily occur two species from *Tetramorium semilaeve* complex (the second one is *Tetramorium punctatum* Santschi) and authors clearly explain the differences between the two species;

Tetramorium semilaeve var. kutteri Santschi, 1927: 57 (tt: Switzerland) – we had no opportunity to study the syntypes of this name but according to the original description this taxon is characterized by dark brown colour, a character never observed in populations of *Tetramorium semilaeve*, and in our opinion it is probably related to (or conspecific with) *Tetramorium diomedeum* Emery or *Tetramorium hungaricum* Röszler, which are the only taxa from this region with dark-brown colour combined with partly reduced head sculpture;

Tetramorium semilaeve var. hoggarense Santschi, 1929b: 103 (tt: Algeria) – we have examined the syntype images available on AntWeb (Available from: https://www.antweb.org/specimen/CASENT0915049) and a sample of workers collected in NE Morocco that well agrees with the syntype morphology. In our opinion it is a distinct species more similar to *Tetramorium sahlbergi* than to *Tetramorium semilaeve*. Its status needs revision based on sexual forms;

Tetramorium semilaeve st. guancha Santschi, 1929a: 150 (tt: Tenerife, =Tetramorium caespitum st. semilaeve var. guancha Santschi, 1921a: 431, unavailable name) – Hohmann et al. (1993) treated this name as a synonym of Tetramorium semilaeve ssp. depressum Forel and we agree with them;

Tetramorium semilaeve subsp. italica Menozzi, 1932: 11 (tt: Italy) – we had no opportunity to study syntypes of this name but according to the original description this taxon is characterized by black colour, a character never observed in populations of *Tetramorium semilaeve*. In our opinion it is probably related to *Tetramorium diomedeum* Emery or *Tetramorium hungaricum* Röszler, the only taxa from this region with dark colour combined with partly reduced head sculpture;

Tetramorium semilaeve subsp. depressiceps Menozzi, 1933: 71 (tt: Palestina) – Collingwood (1985) raised this taxon to the species rank based on material from Saudi Arabia but we are not sure if his interpretation was based on correct identification. Our materials showed than in the Near East there is more than one species from the *Tetramorium semilaeve* complex that is characterized by dark body and the problem needs further study based on all castes;

Tetramorium semilaeve var. jugurtha Menozzi, 1934: 162 (tt: Tunisia, Morocco, Sicily, Dalmatia, =Tetramorium caespitum st. semilaeve var. jugurtha Santschi, 1921a: 430, unavailable name) – Finzi (1940) treated this name as a subspecies of *Tetramorium semilaeve* but undoubtedly this taxon was described from specimens belonging to more than one species. We have examined two syntypes from Tunisia available on AntWeb (Available from: https://www.antweb.org/specimen/CASENT0904819 and https://www.antweb.org/specimen/CASENT0915050) and their characters agree more with *Tetramorium punctatum* than with *Tetramorium semilaeve*. At this moment, we do not have any samples of *Tetramorium semilaeve* from North Africa and Dalmatia. Both *Tetramorium semilaeve* and *Tetramorium punctatum* occur in Sicily and thus the conspecificity of “*jugurtha*” with the true *Tetramorium semilaeve* is not certain;

Tetramorium semilaeve st. syriacum var. cyprium Santschi, 1934: 279 (tt: Cyprus) – this is an unavailable name. Our material of the *Tetramorium semilaeve* complex from Cyprus showed that the Cyprian taxon belongs to species more close to “*galatica*” form rather than the true *Tetramorium semilaeve* (see comment below);

Tetramorium semilaeve var. galatica Menozzi, 1936: 292 (tt: Turkey, =Tetramorium caespitum st. biskrensis var. galatica Santschi, 1921b: 112, unavailable name) – we have examined two syntypes available on AntWeb (Available from: https://www.antweb.org/specimen/CASENT0904820 and https://www.antweb.org/specimen/CASENT0915047) from Turkey and several samples from western Turkey well agree with these syntypes. In our opinion it is a distinct species close to *Tetramorium semilaeve*. Its redescription is under preparation. Probably most records of *Tetramorium semilaeve* from northeastern part of Mediterranean basin refer to the taxon “*galatica*”;

Tetramorium semilaeve subsp. transbaicalense Ruzsky, 1936: 93 (tt: Russia) – Radchenko (1992) synonymized this name with *Tetramorium caespitum*;

Tetramorium semilaeve var. gaetulum Santschi, 1936: 203 (tt: Morocco, =Tetramorium semilaeve st. guancha var. gaetulum Santschi, 1929a: 150 unavailable name) - we have examined syntype available on AntWeb (Available from: https://www.antweb.org/specimen/CASENT0915046) and in our opinion this taxon is more close to *Tetramorium depressum*-*punctatum* complex than to the true *Tetramorium semilaeve*. Its status needs revision based on sexual forms;

Tetramorium semilaeve subsp. atlante Cagniant, 1970: 430 (tt:Tunisia, =Tetramorium caespitum st. punicum var. atlantis Santschi, 1918: 155, unavailable name) – we have examined syntype from Tunisia available on AntWeb (Available from: https://www.antweb.org/specimen/CASENT0915045) and a nest sample with all castes collected in NE Morocco well agrees with the syntype. In our opinion it is a distinct species close to *Tetramorium semilaeve* but distinguished by the morphology of gynes and male genitalia. Its redescription is now under preparation;

*Tetramorium banyulense* Bernard, 1983: 98 (tt: France, Pyrénées-Orientales) – this name was synonymized with *Tetramorium semilaeve* by Casevitz-Weulersse and Galkowski 2009: 497.

From the 22 names listed above, 2 are unavailable for nomenclature, 9 have revised valid status (species or synonym), 2 are under redescription as valid species, and 9 are still difficult to interpretat due to lack of the nest samples with all castes. Our material from Greece and Turkey suggests that some undescribed species of *Tetramorium semilaeve* complex occur in this area and when nest samples with alates become available, they will be described in detail.

## Supplementary Material

XML Treatment for Tetramorium semilaeve
